# Microplastics dysregulate innate immunity in the SARS-CoV-2 infected lung

**DOI:** 10.3389/fimmu.2024.1382655

**Published:** 2024-05-13

**Authors:** Cameron R. Bishop, Kexin Yan, Wilson Nguyen, Daniel J. Rawle, Bing Tang, Thibaut Larcher, Andreas Suhrbier

**Affiliations:** ^1^ Inflammation Biology, QIMR Berghofer Medical Research Institute, Brisbane, QLD, Australia; ^2^ Institut National de Recherche Agronomique, Unité Mixte de Recherche, Oniris, Nantes, France; ^3^ Australian Infectious Disease Research Centre, Global Virus Network (GVN) Center of Excellence, Brisbane, QLD, Australia

**Keywords:** microplastics, SARS-CoV-2, COVID-19, RNA-Seq, inflammation, mouse

## Abstract

**Introduction:**

Global microplastic (MP) pollution is now well recognized, with humans and animals consuming and inhaling MPs on a daily basis, with a growing body of concern surrounding the potential impacts on human health.

**Methods:**

Using a mouse model of mild COVID-19, we describe herein the effects of azide-free 1 μm polystyrene MP beads, co-delivered into lungs with a SARS-CoV-2 omicron BA.5 inoculum. The effect of MPs on the host response to SARS-CoV-2 infection was analysed using histopathology and RNA-Seq at 2 and 6 days post-infection (dpi).

**Results:**

Although infection reduced clearance of MPs from the lung, virus titres and viral RNA levels were not significantly affected by MPs, and overt MP-associated clinical or histopathological changes were not observed. However, RNA-Seq of infected lungs revealed that MP exposure suppressed innate immune responses at 2 dpi and increased pro-inflammatory signatures at 6 dpi. The cytokine profile at 6 dpi showed a significant correlation with the ‘cytokine release syndrome’ signature observed in some COVID-19 patients.

**Discussion:**

The findings are consistent with the recent finding that MPs can inhibit phagocytosis of apoptotic cells via binding of Tim4. They also add to a growing body of literature suggesting that MPs can dysregulate inflammatory processes in specific disease settings.

## Introduction

Global plastic production has grown exponentially, nearly 475 million tons were produced in 2021, and this is set to climb to ≈ 550 million tons in 2026. The latter part of the Anthropocene can now be referred to as the Plasticene (1), with plastic debris providing a new ecological niche known as the plastisphere (2). Plastics degrade into microplastics (MPs), with MP contamination of our environment recognized with mounting concerns (3, 4), especially as chemical breakdown is very slow and biodegradation pathways are limited (5). Human MP exposure arises from both from use of items manufactured from plastic, and as a result of poor disposal of plastic waste that results in plastic pollution (6–8). Less clear are the human health implications of ingestion and inhalation of MPs by general populations, with considerable speculation available (9–12), but a noteworthy paucity of compelling direct evidence for detrimental human clinical outcomes outside heavily contaminated industrial settings. Nevertheless, some compelling clinical data is emerging. For instance, patients with carotid artery plaques in which MPs were detected had a higher risk of a composite of myocardial infarction, stroke, or death (13). A positive correlation has also been reported between faecal MP concentration and the severity of inflammatory bowel disease (14). More MPs were present in the lungs of pediatric patients with severe community acquired pneumonia when compared with those with non-severe community acquired pneumonia (15). MPs were found in cirrhotic liver tissue, but not in liver samples from individuals without underlying liver disease (16). Unfortunately, whether the different MP levels identified in these studies were causative or a consequence of the disease, often remains unclear. More robust evidence for causation is seen for occupational diseases in workers from synthetic textile, flock and (poly)vinyl chloride industries, following exposure to chronic high doses of airborne MPs (17, 18). For instance, chronic interstitial pneumonitis and breathing difficulties have been associated with workplace exposure to nylon flock (19, 20).

Even outside heavily contaminated industrial settings, humans are exposed to airborne MPs at home, at the office and outdoors (21–23), with widespread reports of MPs found in human lungs (15, 24–27) and sputum (28). Estimates from several studies for MP inhalation in the home were 0.3-2.5 µg/kg/d (29). Using indoor dust measurements from 12 countries, the median MP intake was calculated to be 0.36-150 µg/kg/d, with adult intake 10-fold lower than infants (30). An estimated MP inhalation of 6.5-8.97 µg/kg/day is reported to be derived from human exposure models (31–33), although where this data came from or how it was derived is unclear.

Rodent studies seeking to assess the effects of MP inhalation, introduce MPs into the lungs via intranasal or intratracheal inoculation. Unfortunately, as for many such studies on MP exposure, the doses used were often unrealistically high (34). Doses were, for instance, 40 mg/kg/d for 21 days of carboxy-modified 1-5 and 10-20 µm polystyrene beads (35), 5 mg/kg/d for 2 weeks of three types of MPs (33), 5 mg/kg 3 times per week of 100 nm amino-modified polystyrene beads (36), 4 mg/kg (assuming 25 g mouse) 5 µm and 99 nm polystyrene beads every other day for five weeks (37), or 1.25 and 6.25 mg/kg 3 times per week of 5 µm polystyrene beads for three weeks (38). Such doses are 1 to 2 orders of magnitude higher than even the highest estimates for humans. Another serious confounding issue is the potential presence of highly toxic preservatives such as azide, which are commonly added to commercial polystyrene bead products (39). Such chemicals would provide both acute toxicity if the beads are not washed, and would also slowly leach out of the MPs *in vivo*, imbuing the MPs with artificial toxicity. Azide inhibits mitochondrial cytochrome C oxidase (the terminal complex of eukaryotic oxidative phosphorylation) and catalase. Catalase protects cells against oxidative damage by reactive oxygen species (ROS). ROS associated toxicity is frequently reported for MP exposure (40, 41) and may thus simply represent an azide artefact.

An important recent observation is that MPs can be recognized by Tim4, a receptor that plays an essential role in binding and phagocytosing apoptotic cells (a process known as efferocytosis), with MPs able to inhibit efferocytosis by macrophages *in vitro* (42). Azide free, 0.8 µm beads (Sigma-Aldrich, LB8) were used for many of the experiments, with no toxicity or induction of inflammation observed for macrophages treated with MPs *in vitro* (42). Efferocytosis promotes anti-inflammatory activities and inflammation resolution, and efferocytosis failure or efferocytosis defects can result in apoptotic cells undergoing secondary necrosis, which is generally pro-inflammatory *in vivo* (43, 44).

We have previously shown that consumption of azide-free 1 µm polystyrene beads had minimal overt effects by themselves, but after infection with the arthritogenic chikungunya virus, the presence of MPs in the digestive track resulted in a significant prolongation of the ensuing viral inflammatory arthritis (45). Given the SARS-CoV-2 pandemic and the aforementioned estimates on human MP inhalation, we examined herein whether the presence of MPs in the lung would affect the outcome of SARS-CoV-2 infection and COVID-19 disease in a mild non-lethal transgenic mouse model. The transgenic mACE2-hACE2 mouse uses the mouse angiotensin converting enzyme 2 (mACE2) promoter to drive expression of the virus receptor, human angiotensin converting enzyme 2 (hACE2) (46). The inflammatory response and the cytokine signatures associated with COVID-19 are well described (47, 48), and are largely recapitulated in mouse models (46, 49, 50), although these studies used earlier variants of concern. Herein we used a recent SARS-CoV-2 variant of concern, omicron BA.5 (51), with omicron variants currently the dominate SARS-CoV-2 viruses infecting human populations (52). Using this mouse model system and RNA-Seq, we illustrate that a single co-inoculation of MPs and virus into the lungs dysregulated the innate inflammatory response to SARS-CoV-2, initially at 2 days post infection (dpi) suppressing the innate inflammatory responses and later at 6 dpi moving the cytokine response profile towards a “cytokine release syndrome” signature.

## Materials and methods

### Ethics statements and PC3/BSL3 certifications

All mouse work was conducted in accordance with the “Australian code for the care and use of animals for scientific purposes” as defined by the National Health and Medical Research Council of Australia. Mouse work was approved by the QIMR Berghofer Medical Research Institute animal ethics committee (P3600), with infectious SARS-CoV-2 work conducted in a PC3 (Bio-Safety Level 3) facility at the QIMR Berghofer MRI (Australian Department of Agriculture, Water and the Environment certification Q2326 and Office of the Gene Technology Regulator certification 3445). Breeding and use of GM mice was approved under a Notifiable Low Risk Dealing (NLRD) Identifier: NLRD_Suhrbier_Oct2020: NLRD 1.1(a). Mice were euthanized using carbon dioxide.

Collection of nasal swabs from consented COVID-19 patients (to isolate circulating SARS-CoV-2 variants) was approved by the QIMR Berghofer Medical Research Institute Human Research Ethics Committee (P3600).

### The SARS-CoV-2 omicron BA.5 virus isolate

The omicron BA.5 isolate, SARS-CoV-2_QIMR03_ (SARS-CoV-2/human/AUS/QIMR03/2022) belongs to the BE.1 sublineage (GenBank: OP604184.1) and was isolated from a nasal swab, voluntarily collected and donated by a de-identified, consented, adult COVID-19 patient with degree level education (53). Virus stocks were propagated in Vero E6 cells, stocks and tissue culture supernatants were checked for endotoxin (54, 55) and mycoplasma (MycoAlert, Lonza) (56). Virus titres were determined by CCID_50_ assays (57).

### mACE2-hACE2 mice and infection

mACE2-hACE2 mice (58) were generated as described (46) by Monash Genome Modification Platform (MGMP), Monash University and are freely available as heterozygotes through Phenomics Australia (MGMP code ET26). The strain was initially maintained in-house as heterozygotes by backcrossing to C57BL/6J mice. Heterozygotes were then inter-crossed to generate a homozygous mACE2-hACE2 transgenic mouse line. Genotyping was undertaken by digital droplet PCR (MGMP) to distinguish homozygotes from heterozygotes; hACE2 primers 5′-CCAGATGTACCCTCTGCAAG-3′/5′-TCGTGTTCAGGATGGTGTTC-3′, probe 6-carboxyfluorescein-5′-GCTCCAGCTGCAGGCTCTCCAGCA-3′-ZEN/IowaBlack; RPP30 reference primers CTTTGAACTTGTCTATGGTCCT/GCATCAAATTGAGGGCATTG, probe hexachlorofluorescein-TGTGTACCTTCTCATCGTTGCATC-ZEN/IowaBlack. A Bio-Rad QX200 ddPCR droplet generator was used to generate droplets, amplified products were analysed by QX200 droplet reader, and copy numbers were determined using QuantaSoft Analysis Pro Version 1.0 (Bio-Rad, USA). After 2 inter-crossing of homozygotes, all offspring were homozygotes, and a homozygous line was established.

Mice were infected as described (59), briefly, female mice (≈ 10-20 weeks of age) received intrapulmonary infections delivered via the intranasal route with 5×10^4^ CCID_50_ of virus in 50 μl RPMI 1640, while under light anaesthesia. Each group of mice within an experiment had a similar age range and distribution, with the mean age for each group not differing by more than 1 week. Mice were weighed and overt disease symptoms scored as described (53). Mice were euthanized using CO_2_, and tissue titres determined using CCID_50_ assays and Vero E6 cells (57).

### Microplastics

The MPs comprised internally dye loaded, Fluoresbrite^®^ yellow-green polystyrene-based microspheres with a diameter of 1 µm (Cat# 17154-10) purchased from Polysciences as 2.5% w/v in a sterile aqueous suspension without sodium azide. The zeta potential of ≈ 1 µm polystyrene beads has been estimated to be ≈ -20 mV (60). The rationale for choosing these MPs has been described previously (45); briefly, polystyrene MPs are frequently found in the environment, surface labelled microspheres have altered surface characteristics not recapitulate by MPs in the environment (and were thus not used), and the 1 μm size is approximately the size of a bacteria, with bacteria routinely phagocytosed by macrophages. MPs were diluted in PBS and administered alone or together with the viral inoculum in a single dose of 1 µg of MPs per mouse (≈ 40 µg/kg); the total inoculated volume was always 50 µl per mouse.

### MP visualization and quantitation in lung tissues

Lungs were fixed in 10% formalin for 2-3 days, tapped dry and embedded in O.C.T. (Tissue-Tek, Qiagen), with ≈ 7 µm cryosections, under a glass coverslip, viewed by fluorescent microscopy. MPs were counted by eye.

Lungs were weighed, manually chopped using scissors and digested in ammonium sulphate (50 mM), SDS (5 mg/ml) and proteinase K (1 mg/ml) overnight at 37°C as described (61). Digested suspensions were viewed by fluorescent microscopy and a haemocytometer, with fluorescent MPs counted by eye.

### Histology and immunohistochemistry

Histology was undertaken as described (59, 62), with lungs fixed in formalin, embedded in paraffin, sections stained by H&E and slides scanned by Aperio AT Turbo (Aperio, Vista, CA, USA). Image analysis (nuclear/cytoplasmic staining ratios) was undertaken using Positive Pixel Count v9 algorithm. White space analysis was undertaken using QuPath v0.2.3.

Immunohistochemistry (IHC) was undertaken as described (63) using the macrophage/monocyte monoclonal antibody F4/80 (Abcam, Cambridge, MA) and color developed using NovaRed (Vector Laboratories, Newark, CA, USA).

### RNA-Seq and bioinformatic analyses

RNA-Seq and bioinformatic analyses were undertaken as described (46, 59). Briefly, mouse lung tissues were harvested into RNAlater, RNA was extracted using TRIzol (Life Technologies), and RNA concentration and quality measured using TapeStation D1kTapeScreen assay (Agilent). cDNA libraries were generated using Illumina TruSeq Stranded mRNA library prep kit and sequencing performed in-house using Illumina Nextseq 2000 platform (75-base paired end reads). Processed reads were aligned to GRCm39 vM31 (mouse genome) and the BA.5 genome using STAR aligner. Gene expression was calculated using RSEM and EdgeR. For the BA.5+MP vs. BA.5 data sets a term for viral reads was introduced into EdgeR to minimize the effects of viral loads on significance and fold change in the mRNA expression data. A filter was applied to the count matrix of counts per million (cpm) >1 for any given gene in at least 5 samples.

Differentially expressed genes (DEGs) were analysed using Ingenuity Pathway Analysis (IPA) (QIAGEN). Whole gene lists ranked by fold change were interrogated using Gene Set Enrichment Analyses (GSEA v4.0.3) (Broad Institute, UCSanDiego) using the “GSEAPreranked” module. Gene sets were obtained from the complete Molecular Signatures Database (MSigDB) v7.2 (31,120 gene sets) (msigdb.v7.2.symbols.gmt), Blood Transcription modules (BTMs) (64), and Xue at al., 2014 (65). Relative abundance of specific cell types was estimated via cellular deconvolution using the SpatialDecon package in R (66), with cell-type expression matrices obtained from Yoshida et al., 2019 (67) or the NanoString Cell Profile Library, either Mouse/Adult/Lung_MCA (Mouse cell atlas) or Mouse/Adult/ImmuneAtlas_ImmGen_cellfamily (Immune cell family) (available at https://github.com/Nanostring-Biostats/CellProfileLibrary/tree/master/Mouse/Adult).

Kraken metagenomic sequence classification was undertaken as described (68). The Interferome database (69) was used to identify interferon regulated genes among DEG sets.

### RT-qPCR

Total RNA was used to synthesize cDNA using ProtoScript II First Strand cDNA Synthesis Kit (New England Biolabs) and qPCR performed using iTaq Universal SYBR Green Supermix (Bio-Rad) as per manufacturer’s instructions with primers Nr4a1 (Forward 5’-GTTGGGGGAGTGTGCTAGA-3’ and Reverse 5’-AATACAGGGCATCTCCAGCC-3’), Ccn1 (Forward 5’-AAGAGGCTTCCTGTCTTTGGC-3’ and Reverse 5’- AACTCGTGTGGAGATGCCAG-3’), Hspa1a (Forward 5’-TTTGTGTATTGCACGTGGGC-3’ and Reverse 5’-CCAGGGGAGAGTCCAAACAC-3’), Hspa1b (Forward 5’- AATGTTGGGAGCAGCACTGT-3’ and Reverse 5’-TGTCTTCCCAGGCTACTGGA-3’), Oas3 (Forward 5’-TGGCAATCCCATCAAGCCAT-3’ and Reverse 5’- CTGAGGGCTGGTGTCACTTT-3’), Irf7 (Forward 5’-ACCGTGTTTACGAGGAACCC-3’ and Reverse 5’-GTTCTTACTGCTGGGGCCAT-3’), Ccl4 (Forward 5’- GCCAGCTGTGGTATTCCTGA-3’ and Reverse 5’-TGAACGTGAGGAGCAAGGAC-3’). qPCR reactions were performed in duplicate and averaged. Gene expression was normalised using mRPL13a as the house-keeping gene (70). Log_2_ fold-change was calculated using the 2-ΔΔCt method (71).

### Statistics

Statistical analyses of experimental data were performed using IBM SPSS Statistics for Windows, Version 19.0 (IBM Corp., Armonk, NY, USA). The t-test was used when the difference in variances were <4 fold (determined using Data Analysis ToolPak in Excel), skewness was >-2 and kurtosis was <2 (determined using SPSS). Otherwise, the non-parametric Kolmogorov-Smirnov asymptotic test was used (SPSS).

## Results

### MP inoculation into mouse lungs promotes mild inflammation on day 2

Experimental delivery of MPs into mouse lungs generally involves delivery of MPs in suspension into the lungs via the intranasal (i.n.) route (33, 35–38), as mimicking MP dust inhalation is currently technically, logistically and ethical difficult in a laboratory setting. Some of the challenges include consistent airborne MP dosing, staff safety considerations, and mouse eye irritation, respectively.

**Figure 1:**
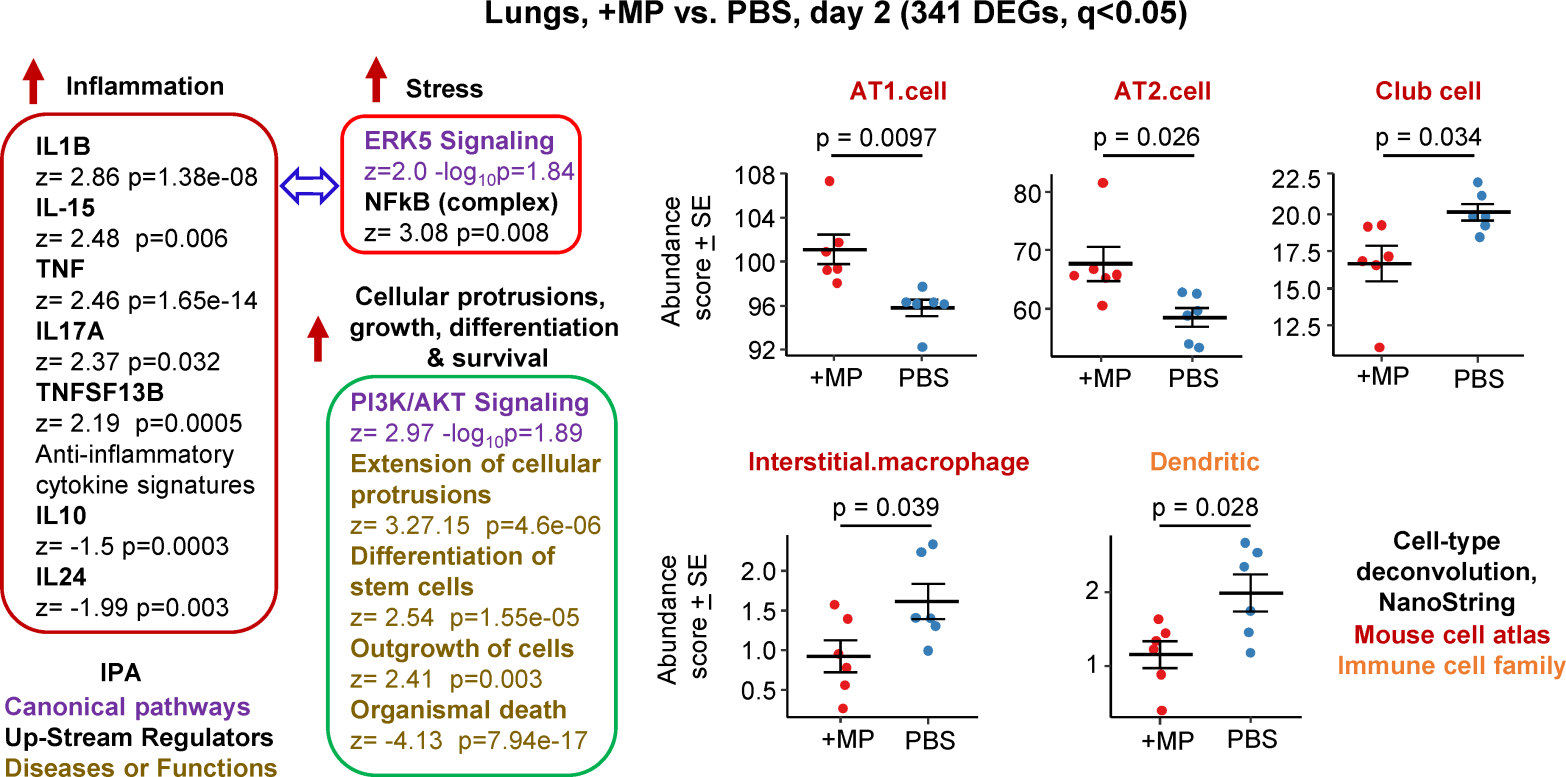
Bioinformatic analyses of RNA-Seq data for lungs inoculated with MPs. C57BL/6J mice were inoculated with MPs or PBS into the lungs via the intranasal route and at 2 days post inoculation lungs were harvested and analysed by RNA-Seq. DEGs (n=341) were identified and analysed by IPA, with selected annotations shown, full data sets are provided in **Supplementary Table 1**. The whole gene list (**Supplementary Table 1**) was also analysed by cell type deconvolution using cell-type expression matrices obtained from NanoString; statistics by t tests.

To assess the effects of MPs on uninfected lungs, a single dose of 1 µg of MPs per mouse (≈ 40 µg/kg) of azide-free, 1 µm diameter, fluorescent dye loaded, polystyrene beads (density 0.26 g/ml) was delivered in 50 µl of PBS via the i.n. route into the lungs of lightly anesthetized female C57BL/6J mice. Control mice received 50 µl of PBS. The anaesthesia prevents the mouse sneezing or coughing out the introduced material, but is light enough for the mouse to retain largely normal breathing, promoting deep lung delivery of the material; this is the same method used to infect mice with SARS-CoV-2 (46, 51, 59, 72, 73). The mice were observed daily and no overt clinical signs were observed. Mice were euthanized on day 2 and 6 post MP inoculation, and lungs analysed by RNA-Seq, with bioinformatic treatments comparing +MP day 2 vs. PBS day 2 and +MP day 6 vs. PBS day 6 (**Figure 1**; **Supplementary Tables 1**, **2**).

RNA-Seq for +MP vs. PBS day 2 identified 341 differentially expressed genes (DEGs) at q (FDR) <0.05 (**Supplementary Table 1**). Fold change (FC) was low overall, with only 9 genes showing FC>2 (log2 FC>1) and only 4 of these had a mean normalized counts per million (cpm) >10 across all samples (**Supplementary Table 1**). Ingenuity Pathway Analysis (IPA) indicated a mild pro-inflammatory response, with IL-1β the highest Cytokine UpStream Regulator (USR) by z score (**Figure 1**, Inflammation; **Supplementary Table 1**). Some signatures that can be associated with stress responses were also evident, principally ERK5 (MAPK7) (74, 75) and NFkB (76, 77) (**Figure 1**, Stress). A series of annotations associated with cellular protrusions, proliferation, differentiation and survival were identified as the top annotations (by z score) by IPA Diseases and Functions (**Figure 1**), with the largest negative z scores associated with growth failure or death (**Supplementary Table 1**).

Relative abundance of specific cell types was analysed via cellular deconvolution, SpatialDecon (66), with cell-type expression matrices obtained from Yoshida et al., 2019 (67) or the NanoString Cell Profile Library, either Mouse/Adult/Lung_MCA (Mouse cell atlas) or Mouse/Adult/ImmuneAtlas_ImmGen_cellfamily (Immune cell family) (**Supplementary Table 1**). These analyses suggested that the cells that were more transcriptionally active (expanding/repairing) in the +MP group were AT1 and AT2 type I and II alveolar epithelial cells (**Figure 1**, AT1, AT2). The positive z score for “Differentiation of stem cells” (**Figure 1**, Cellular protrusions, growth, differentiation, survival), may be associated with reduced abundance of Club cells (formally known as Clara cells), which are regional progenitor cells that repair bronchiolar epithelium in response to lung damage. Also identified was a reduced abundance of interstitial macrophages, cells that are associated with nerves and airways and appear to have important immunoregulatory roles (78, 79). A reduction in interstitial macrophages may also be consistent with the reductions in anti-inflammatory cytokines (**Figure 1**, IL-10 and IL-24) (79). Dendritic cell abundance was also down in the +MP group, perhaps consistent with mobilization to lymph/lymph nodes in response to inflammation (80).

In summary, MP inoculation into lungs generated a transcriptional signature indicating a mild inflammatory response, and mild increases in abundance scores (likely proliferation) for lung epithelial cells, and reductions in abundance scores for interstitial macrophages and dendritic cells.

### Effects of a single MP inoculation into lungs are largely resolved within 6 days

Harvesting of lungs on day 6 after MP or PBS inoculation and comparing the transcriptome by RNA-Seq (+MP day 6 vs. PBS day 6) identified only 7 DEGs, all with low fold change (**Supplementary Table 2**). This argues that the lung response to a single exposure to MPs is largely resolved within 6 days. GSEAs using MSigDB gene sets provided a series of significant annotations with high positive Normalised Enrichment Score (NES) associated with epithelial cells (e.g. Epithelial differentiation), suggesting some lung repair activities were still underway on day 6 (**Supplementary Table 2**).

### The mACE2-hACE2 omicron BA.5 mouse model of SARS-CoV-2 infection and disease

The best described mouse model for SARS-CoV-2 infection is the K18-hACE2 model which expresses the SARS-CoV-2 receptor, human ACE2 (hACE2), from the keratin 18 promoter (K18). This model is generally lethal within several days, as it usually leads to brain infection and ensuing weight loss that reaches ethically defined criteria for euthanasia (53, 59, 73). A non-lethal, less severe model is the mACE2-hACE2 model, wherein hACE2 is expressed from the mouse ACE2 promoter (mACE2) (58). We generated such a transgenic mouse on a pure C57BL/6J background by microinjection of the mACE2-hACE2 transgene into the pronucleus of C57BL/6J zygotes at the pronuclei stage (46) and generated a homozygous mACE2-hACE2 transgenic mouse line (see Materials and Methods). Given MP-mediated effects were generally mild (**Figure 1**; **Supplementary Table 1**), we chose this mouse model so that any MP-mediated perturbations might be more readily detected.

Although infection of mACE2-hACE2 mice with an original strain isolate has been described (46, 58), infection of homozygous mACE2-hACE2 mice with an omicron BA.5 isolate has not. The latter did not result in significant weight loss (data not shown), with lung histology at 6 days post infection (dpi) showing a series of histopathological features that have been described previously in COVID-19 mouse models (49, 58, 72) (**Supplementary Figure 1**). Lesions were less severe when compared with those seen after infection of K18-hACE2 mice with an original strain isolate (59, 81, 82), although significant loss of white space (unstained air-spaces) in H&E stained lung sections (indicating lung consolidation) (51, 72), was also seen in this model (**Supplementary Figure 2**).

Infection of mACE2-hACE2 mice with BA.5 (BA.5 vs. PBS) was analysed by RNA-Seq, with lungs harvested on 2 dpi (peak viral load) and 6 dpi (peak lung pathology) and compared with mock infected lungs (PBS) harvested on days 2 and 6, respectively (**Supplementary Tables 3**, **4**). When the lung cytokine response signatures (IPA Cytokine USR z scores) from BA.5-infected mACE2-hACE2 mice, were compared with those from K18-hACE2 mice infected with an original strain isolate (46), a highly significant correlation emerged for 2 dpi. However, at 5/6 dpi a less significant correlation was observed (**Supplementary Figure 3**), reflecting the lower severity of lung disease in the BA.5 mACE2-hACE2 model.

IPA Diseases and Function annotations for BA.5 infected lungs for mACE2-hACE2 mice (BA.5 vs. PBS) pertinent to the analyses below include, top annotations for leukopoiesis/haematopoiesis, phagocytosis (engulfment of cells) and apoptosis & necroptosis (**Supplementary Tables 3**, **4**).

### MP clearance from lungs is slower in BA.5 infected mice

**Figure 2:**
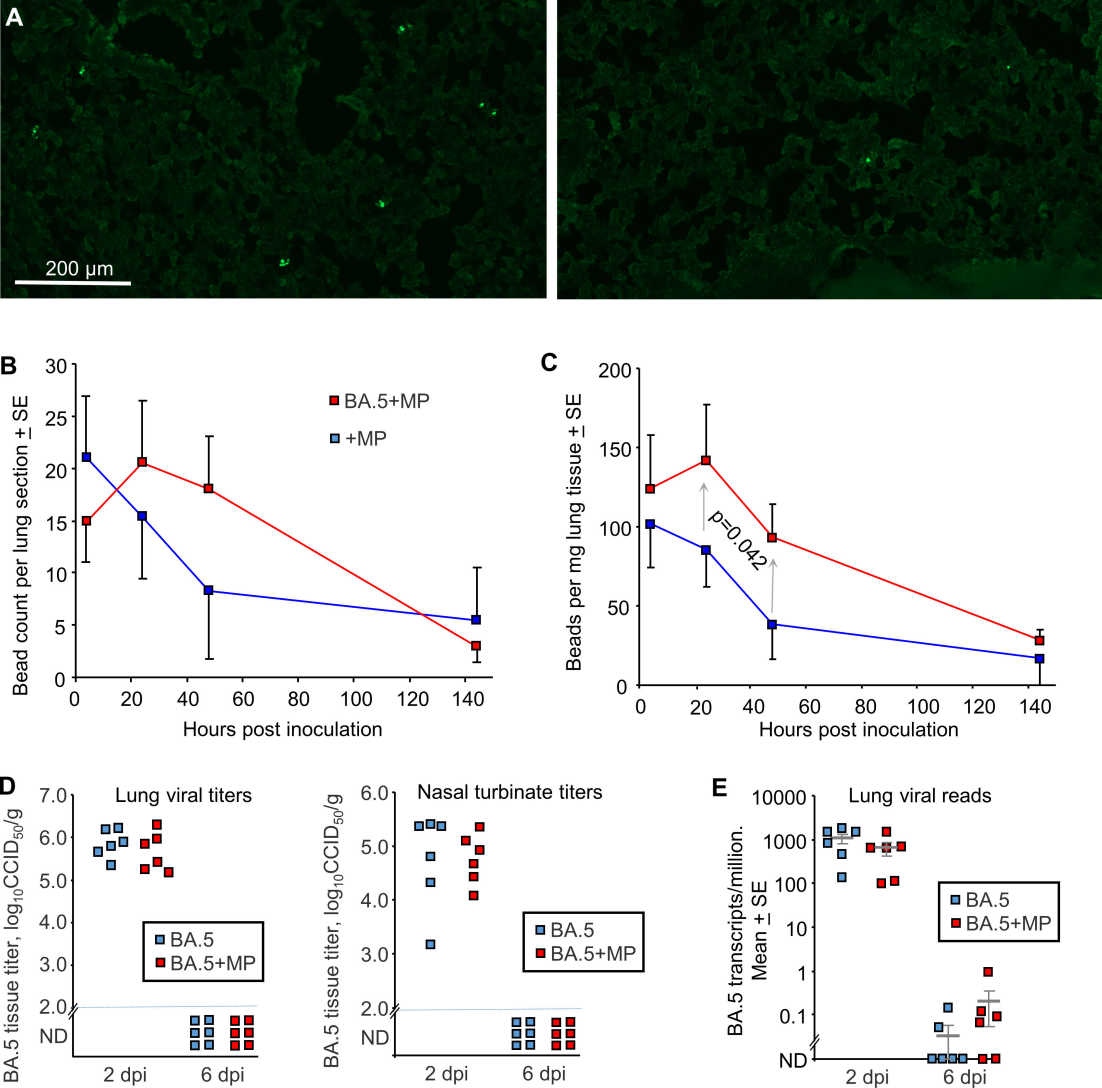
SARS-CoV-2 infection slowed MP clearance, but MPs did not affect viral load. **(A)** mACE-hACE2 mice were inoculated with BA.5+MP or with just MP (+MP), lungs were harvested at different time points and cryosections of lungs observed by fluorescent microscopy. Examples are shown for the 4 hr (right) and 48 hr (left) time points for the +MP group. **(B)** Quantitation of MP counts from cryosections (n=6 mice per group for 4 hrs, n=3 for other time points). **(C)** Quantitation of MP counts using tissue digestion and hemocytometer. The two groups were statistically different by 2 way ANOVA, which included a term for hours post inoculation with data for 24 and 48 hrs included. **(D)** mACE-hACE2 mice were inoculated with BA.5+MP or with BA.5 (no MP) and lung and nasal turbinate tissue titres determined by CCID_50_ assays. **(E)** mACE-hACE2 mice were inoculated with BA.5+MP or with BA.5 and lung analysed by RNA-Seq. Viral read counts are shown as BA.5 counts per million.

To evaluate the effects of SARS-CoV-2 infection on MP clearance from lungs, mACE2-hACE2 mice were given a single inoculum of 50 µl containing both BA.5 (5x10^4^ CCID_50_) and 1 µg (≈40 µg/kg) of azide free MP beads into the lungs via the intranasal route using the same procedure used herein and generally to infect mice with SARS-CoV-2 (59, 72, 83) (BA.5+MP). Control mice were not infected, but received the same 50 µl inoculum containing MP (+MP). Mixing the beads with the viral inoculum prior to infection prevented any complications that might arise from simple liquid occlusion of airways, if for, instance, the SARS-CoV-2 inoculum was given first and MPs were introduced later. Lungs were harvested at ≈ 4 hrs, 24 hrs, 48 hrs (day 2) and 144 hrs (day 6), were fixed in formalin, and the beads (MPs) observed by fluorescent microscopy of cryosections (**Figure 2A**). Quantitation suggested slower clearance of the MPs in BA.5-infected lungs (**Figure 2B**), consistent with SARS-CoV-2-mediated disruption of the ciliary layer, which is responsible for mucociliary clearance (59, 84). To provide better quantitation, lungs were digested in proteinase K and dissolved in SDS, and beads counted using a hemocytometer under a fluorescent microscope. The same trend was observed, with significantly more beads present at 24 and 48 hrs in infected mice (**Figure 2C**). Results from both assays indicated that MPs were largely cleared by day 6 (**Figures 2B, C**).

### Lung viral loads were unaffected by MPs

To evaluate the effects of MPs on SARS-COV-2 infection, mACE2-hACE2 mice were given a single inoculum of 50 µl containing both BA.5 (5x10^4^ CCID_50_) and 1 µg (≈40 µg/kg) of beads, delivered into the lungs via the intranasal route (BA.5+MP). Control mice received the same 50 µl inoculum containing BA.5, but no MPs (BA.5). Lungs and nasal turbinates were harvested on 2 and 6 days post infection (dpi) and tissue titres determined by CCID_50_ assays, with no significant differences in viral titres evident on 2 dpi, and below the level of detection by 6 dpi (**Figure 2D**).

Lungs were also analysed by RNA-Seq and reads aligned to the viral genome, with the number of viral reads not significantly different for groups with or without MPs at 2 or 6 dpi (**Figure 2E**; **Supplementary Tables 5**, **6**). Thus overall MP inoculation had no significant effects on viral loads in the respiratory track.

### MPs reduced innate proinflammatory signatures in BA.5 infected lungs 2 dpi

**Figure 3:**
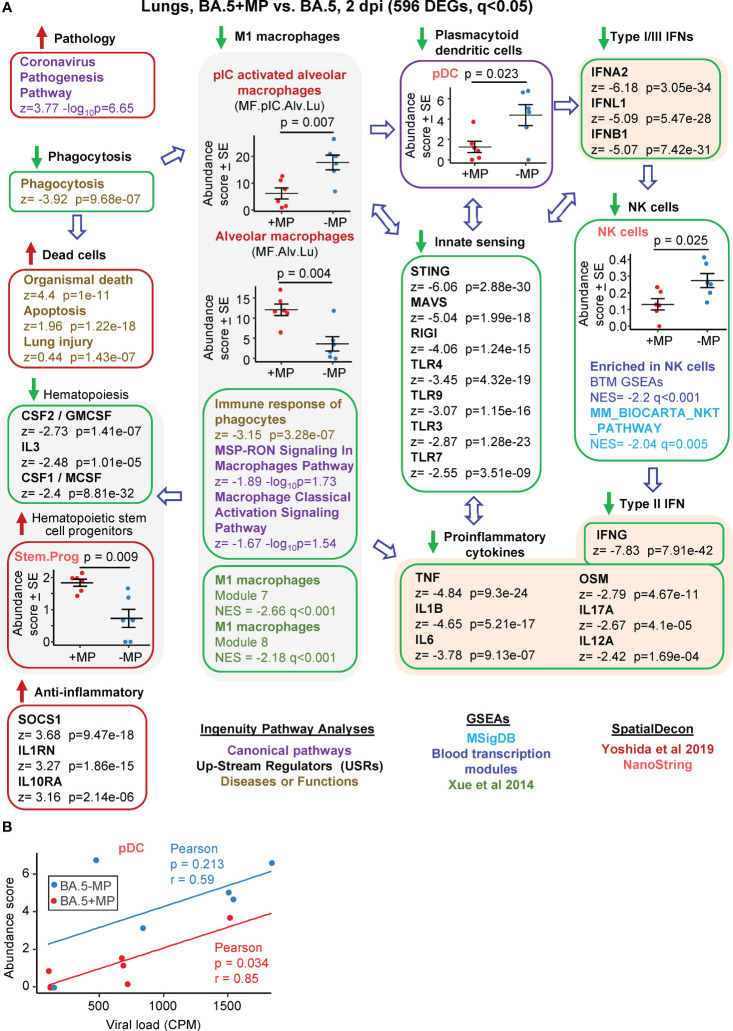
Bioinformatic analyses of RNA-Seq data for BA.5+MP vs. BA.5 at 2 dpi. **(A)** mACE-hACE2 mice were inoculated with BA.5+MP or with BA.5 and at 2 dpi lungs were harvested and analysed by RNA-Seq. 596 DEGs were identified and analysed by IPA, with selected annotations shown; full data sets are provided in **Supplementary Table 5**. The whole gene list was also analysed by GSEAs, and cellular deconvolution (SpatialDecon) using cell-type expression matrices obtained from Yoshida et al., 2019 and NanoString (statistics by t tests). **(B)** Cellular deconvolution data for plasmacytoid dendritic cells shown in ‘a’ plotted against viral reads (counts per million). Pearson correlation significance (p) and correlation coefficient (r) provided.

To assess the effects of MPs on the innate immune responses induced by BA.5 infection, the same groups described above (BA.5+MP vs. BA.5, 2 dpi) were compared by RNA-Seq, with reads aligned to the mouse genome. To minimize within-treatment variation in gene expression due to variability in viral load (**Figure 2E**; **Supplementary Table 5**), viral loads expressed in counts per-million were included as a term in the linear model used for estimating gene expression in EdgeR. Applying a q<0.05 filter, 596 DEGs were thereby identified (**Figure 3**; **Supplementary Table 5**).

The DEGs were analysed by IPA as above, and the ‘All gene’ list, ranked by fold change, was used in a series of Gene Set Enrichment Analyses (GSEAs) using gene sets from the Molecular Signatures Data Base (MSigDB), Blood Transcription modules (BTM) and Xue et al., 2014 (65) (**Figure 3**; **Supplementary Table 5**). Relative abundance of specific cell types was analysed as above using SpatialDecon (66), using gene expression matrices provided by NanoString Cell Profile Library, and Yoshida et al., 2019 (67) (**Figure 3**; **Supplementary Table 5**).

The highest ranked IPA Canonical pathway was the ‘Coronavirus pathogenesis pathway’ (**Figure 3A**, Pathology; **Supplementary Table 5**). MPs were recently reported to inhibit phagocytosis of apoptotic cells (known as efferocytosis) (42), and a top IPA Diseases and Functions annotation was ‘Phagocytosis’, with a high negative z score (**Figure 3A**, Phagocytosis; **Supplementary Table 5**). During infection, macrophages phagocytose SARS-CoV-2-infected cells and are thereby activated, and in turn mediate strong activation of plasmacytoid dendritic cells (pDC) (85). The reduced phagocytosis is thus consistent with a reduction in activated M1 macrophages identified via a series of annotations (**Figure 3**, M1 macrophages); specifically (i) reduced abundance of polyinosinic:polycytidylic acid (pIC) stimulated macrophages (a stimulus that mimics viral double stranded RNA), (ii) a series of IPA Canonical pathways and Diseases and Functions macrophage annotations with negative z scores, and (iii) GSEAs with negative NES and q<0.05 using M1 macrophage gene sets from Xue at al., 2014 (65) (**Supplementary Table 5**).

Significantly reduced numbers of pDC were identified in the BA.5+MP group by cellular deconvolution using NanoString expression matrices (**Figure 3**, pDC, **Supplementary Table 5**), consistent with the reduction in M1 macrophages (85). As might be expected, viral load and pDC abundance showed positive correlations, with pDC abundance lower for the BA.5+MP group across all viral loads (**Figure 3B**). pDC are dominant producers of type I interferons (IFNs) during SARS-CoV-2 infection, with low numbers of pDCs and low type I IFN levels generally associated with increased COVID-19 severity (86, 87). Consistent with reduced abundance of pDC in the BA.5+MP group, was a series of type I IFN IPA USR annotations with highly negative z scores (**Figure 3A**, Type I IFNs; **Supplementary Table 5**). Reduction in type I IFN signatures was also evident from GSEAs using MSigDB gene sets (**Supplementary Table 5**). The general overall reduction in innate sensing signatures (**Figure 3A**, Innate sensing) was consistent with reduced type I IFN signatures, as well as the signatures associated with reduced phagocytosis, M1 macrophages and pDCs.

The lower type I and type III IFN signatures in the BA.5+MP group were not associated with significantly higher viral titres or higher levels of viral RNA (**Figures 2D, E**). This might be expected as even elimination of the type I or type III IFN receptor (in IFNAR^-/-^ and IL-28RA^-/-^ mice, respectively) had no significant impact on viral replication in the lungs (49). The ability of multiple SARS-CoV-2 proteins to mediate evasion of innate IFN responses is well described (88, 89), and explains the insensitivity of the virus to these responses. SpatialDecon, and GSEAs using MSigBD and Blood Transcription modules (BTMs), identified significantly reduced Natural Killer (NK) cell signatures in the BA.5+MP group (**Figure 3A**, NK cells; **Supplementary Table 5**), with low early type I IFN levels linked to reduced NK activity during viral infections generally (90) and likely also SARS-CoV-2 infections (91). NK cells have the capacity to exert important early innate antiviral activities; however, SARS-CoV-2 shows a remarkable ability to evade this arm of the immune system (92). NK and NKT cells are important sources of early IFNγ (90), thus the high negative z score for the IPA USR annotation for IFNγ (**Figure 3A**, Type I IFN; **Supplementary Table 5**) is consistent with reduced abundance of NK and NKT cells.

The general reductions in innate immune signatures for BA.5+MP vs. BA.5 (**Figure 3**, Innate sensing, Type I IFNs, NK cells) is likely to be responsible for the negative z scores for a series of IPA USR pro-inflammatory cytokine annotations (**Figure 3A**, Proinflammatory cytokines; **Supplementary Table 5**). Overall, these data illustrate that MPs can significantly ameliorate SARS-CoV-2-mediated activation of innate immune responses in the lung at 2 dpi.

### RT-PCR confirmation of down-regulation of interferon regulated genes

The Interferome database provides an open-access bioinformatic resource that allows identification of interferon regulated genes (IRGs). The 100 most down-regulated DEGs at 2 dpi (according to EdgeR) were interrogated using the Interferome database, with a total of 45 of these identified as type I IRGs (**Supplementary Table 5**). Three of these (Ccl4, Irf7, and Oas3) were among the most down-regulated DEGs by RNA-Seq. Their significant differential expression was validated by RT-qPCR (**Supplementary Figure 4A**).

### Haematopoietic stem cell progenitors are elevated by MPs 2 dpi

Three cytokine signatures associated with haematopoiesis were identified as down-regulated in the IPA USR analysis; CSF1/M-CSF, CSF2/GM-CSF and IL3 (**Figure 3**, Haematopoiesis; **Supplementary Table 5**). Cellular deconvolution using SpatialDecon and the Nanostring expression matrices also identified a higher abundance of haematopoietic stem and progenitor cells (HSPC) in the lungs of the BA.5+MP group at 2 dpi (**Figure 3A**, Stem.Prog; **Supplementary Table 5**). M-CSF, GM-CSF and IL3 (as well as IFNα, IFNγ and IL1, and TLR) are some of the key cytokine signaling pathways that promote HSPC differentiation (93). These analyses thus suggest an accumulation of undifferentiated HSPC due to reduced differentiation of HSPC into myeloid and/or lymphoid precursors.

Dysregulated emergency myelopoiesis and immature myeloid cells are associated with poor outcomes in COVID-19 patients (94–96), although these are observations from peripheral blood during COVID-19 disease, rather than from lungs early post infection. Nevertheless, these data (**Figure 3A**, Haematopoiesis & Haematopoietic stem and progenitor cells) suggest MPs in the lung suppress haematopoiesis, likely as a result of the overall reduction in the proinflammatory milieu for BA.5+MP. To clarify the terminology used in these annotations; haematopoiesis includes myelopoiesis, lymphopoiesis and erythropoiesis, whereas leukopoiesis encompasses myelopoiesis and lymphopoiesis.

### Some anti-inflammatory signatures increased by MPs at 2 dpi

IPA analysis of BA.5+MP vs. BA.5 indicated some signatures that are usually associated with anti-inflammatory activity (**Figure 3A**, Anti-inflammatory). The high USR z score for TRIM24 (**Supplementary Table 5**) argues that, although M1 macrophages are down, M2 macrophages are not increased, given that TRIM24 expression is suppressed in M2 macrophages (97).

Interstitial macrophages can have immunosuppressive properties (79, 98, 99); however, although identified as down in **Figure 1** they were not identified as increased for BA.5+MP vs. BA.5. Conceivably, these anti-inflammatory signatures arose from the higher abundance of alveolar macrophages that are not M1 biased.

### MPs promote some proinflammatory signatures at 6 dpi

**Figure 4:**
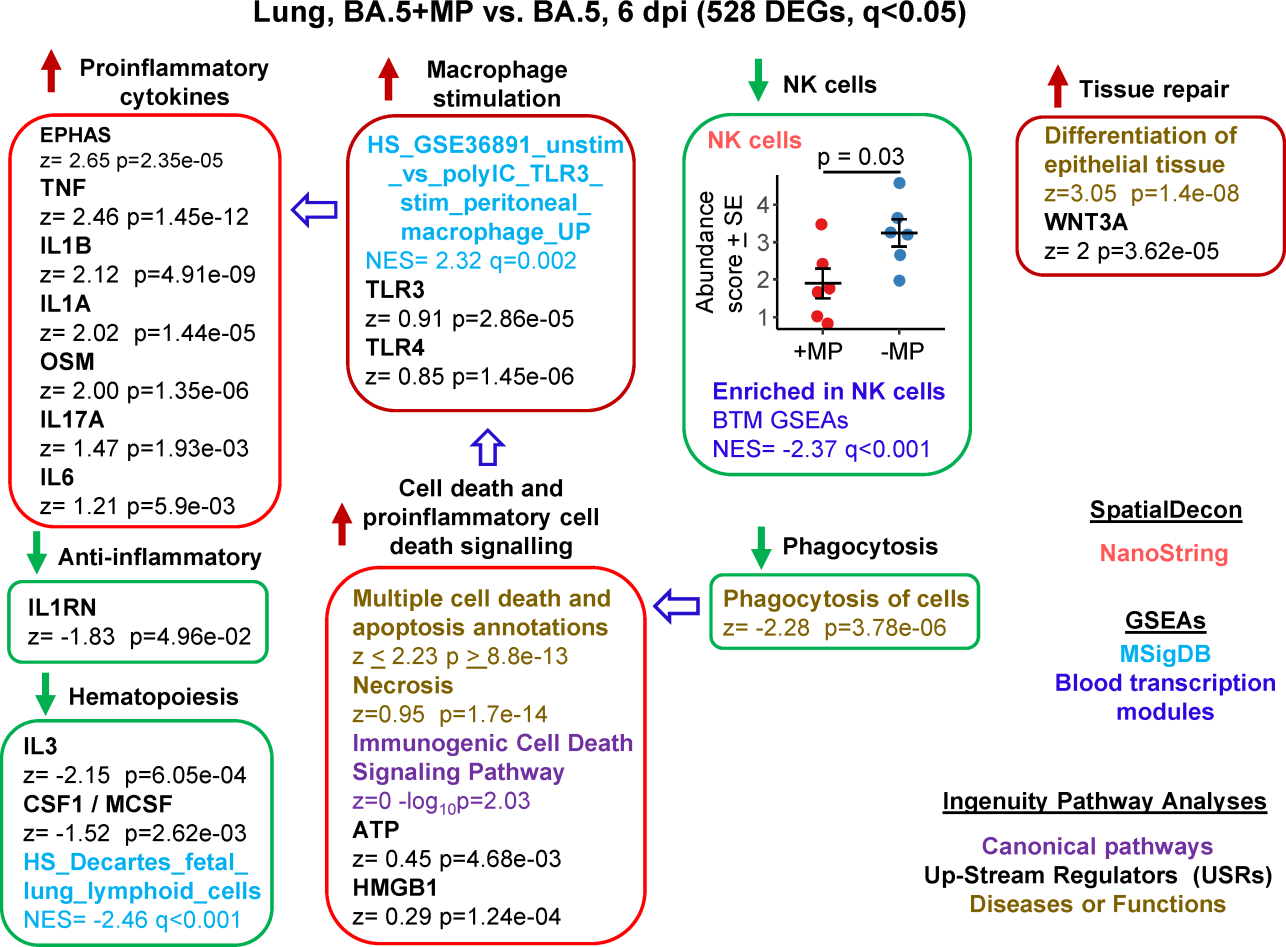
Bioinformatic analyses of RNA-Seq data for BA.5+MP vs. BA.5 at 6 dpi. mACE-hACE2 mice were inoculated with BA.5+MP or with BA.5 and at 6 dpi lungs were harvested and analysed by RNA-Seq; 528 DEGs were identified. Bioinformatic analyses as in **Figure 3**. Full data sets are provided in **Supplementary Table 5**.

RNA-Seq was also undertaken for BA.5+MP vs. BA.5 at 6 dpi (the day of peak pathology in this model) with reads again aligned to the mouse genome. Using the same filter (q<0.05) 528 DEGs were identified and were analysed as above (**Supplementary Table 6**). A number of proinflammatory cytokine IPA USR signatures were up-regulated (**Figure 4**, Proinflammatory cytokines), all are associated with increased COVID-19 severity; TNF (100, 101), IL1B (102), IL1A (103), OSM (104), IL17A (105), and IL6 (106). Upregulated activity of the transcription factor EPHAS is also associated with pulmonary inflammatory responses in lethal COVID-19 (107). The anti-inflammatory interleukin 1 receptor antagonist (IL1RN) signature was down-regulated, with reduced IL1RN recently associated with increased COVID-19 severity (108).

The increased proinflammatory cytokine signatures (**Figure 4**) were not associated with reduced viral loads (**Figure 2E**). Of these responses, TNF is described as having anti-viral activity in some settings (109–111). However, anti-TNF therapy has not been associated with increases in SARS-CoV-2 replication or COVID-19 severity (100, 112), suggesting that TNF has minimal anti-viral activity against SARS-CoV-2 during COVID-19.

The mechanisms responsible for the increase in proinflammatory signatures (**Figure 4**) remain unclear, with, for instance, reduced type I responses (**Figure 3**) previously associated with reduced (not increased) lung inflammation (49). However, GSEAs using MSigDB gene sets provided a significant macrophage annotation with high NES for TLR3 stimulation, with an IPA USR annotation for TLR3 signaling also identified, albeit with a relatively low z score (**Figure 4**, Macrophage stimulation). Viral double-stranded RNA is a probable ligand for TLR3 (113). The reduced phagocytosis (**Figure 4**, Phagocytosis), and the increased proinflammatory responses (**Figure 4**, Proinflammatory cytokines), might suggest increased secondary necrosis due to a reduction in the phagocytosis of apoptotic cells (efferocytosis) (114–116). Multiple IPA Disease & Functions annotations suggest an increase in apoptosis signatures (**Supplementary Table 6**), with secondary necrosis known to be proinflammatory via secretion of a number of mediators such as HMGB1 and ATP (114). These latter mediators were identified as IPA USRs, although again with relatively low z scores (**Figure 4**, Cell death and proinflammatory cell death signaling).

NK cell and haematopoiesis signatures remained, as at 2 dpi, down-regulated by MPs (**Figure 4**). Tissue repair signatures were also identified (**Figure 4**, Tissue repair) as might be expected by 6 dpi, when virus has been largely cleared (**Figures 2D, E**).

### MPs promote a ‘cytokine release syndrome’ profile in BA.5 infected lungs at 6 dpi

**Figure 5:**
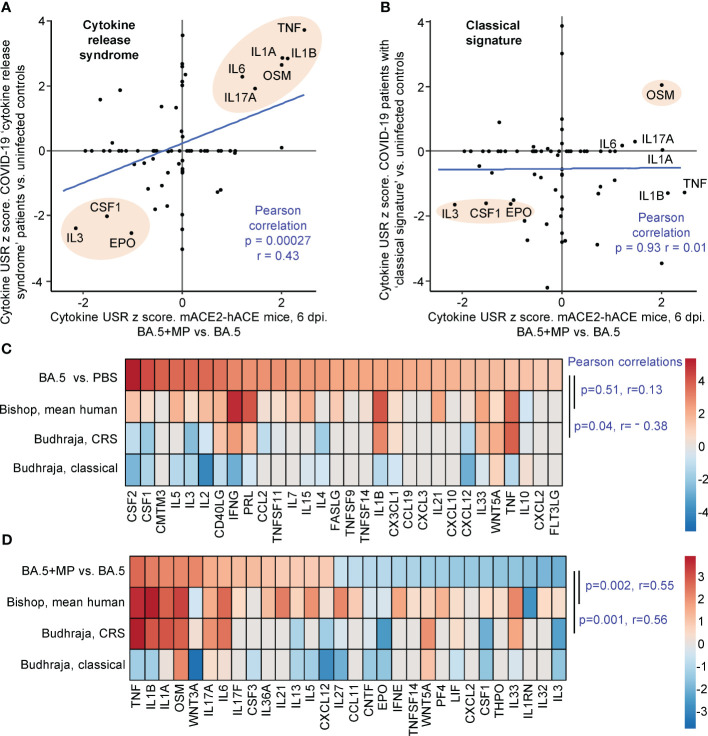
IPA cytokine USR z score correlations between human studies and BA.5+MP vs. BA.5 at 6 dpi. **(A, B)** IPA Cytokine USR z scores from the IPA analysis of the 528 DEGs described in **Figure 4** were plotted against IPA Cytokine USR z scores from the IPA analysis of DEGs generated from fastq files obtained from NCBI SRA Bioproject PRJNA761132 (Budhraja et al., 2022) (**Supplementary Table 7**). Two distinct patterns were described for severe COVID patients, ‘Cytokine release syndrome’ and ‘Classical signature’; Pearson correlation significance (p) and correlation coefficient (r) are provided for both. Pink shading shows USRs dominant (high z scores) in both human and mouse studies. **(C)** The top 30 cytokine USRs by absolute z scores for BA.5 vs. PBS are compared to the human lung cytokine USR z scores described in Bishop et al., 2022 (derived from 4 studies of SARS-CoV-2 infected vs. uninfected), and the cytokine USR z scores for ‘Cytokine release syndrome’ and ‘Classical signature’ described in Budhraja et al., 2022 [same as **(A, B)** above]. **(D)** The top 30 cytokine USRs by absolute z scores for BA.5+MP vs. BA.5 are compared as for **(C)**. Pearson correlation significance (p) and correlation coefficient (r) are provided.

Human lung RNA-Seq data for severe lethal COVID-19 infections was recently provided, with two signatures described, a ‘Classical signature’ and a ‘cytokine release syndrome’ (CRS) signature (117). We re-derived two DEG lists from the fastq files deposited for this study (**Supplementary Table 7**; PRJNA1036279), with the lists then analysed by IPA. Significant cytokine USR z scores provided by this analysis were compared with the z scores of significant cytokine USRs identified for BA.5+MP vs. BA.5 (**Figures 5A, B**; **Supplementary Table 7**). A significant correlation emerged for CRS (**Figure 5A**). In both human (infected vs. uninfected) and mouse (BA.5+MP vs. BA.5) data sets, TNF, IL1A, IL1B, OSM, IL6 and IL17 signatures were prominently up-regulated, and CSF1, IL3 and EPO were prominently down-regulated (**Figure 5A**, pink shading). These cytokines and their association with severe COVID-19 are described above for **Figure 4**. Treatment with EPO (118) and CSF1 (119, 120) have been considered for COVID-19, with low IL-3 levels associated with increased severity (121).

Budhraja et al., 2022 also described a “Classical signature” as the more common pattern for lethal COVID-19 (117); a similar IPA analysis indicated no correlation when cytokine USR z scores for the ‘Classical signature’ were compared with cytokine USRs z scores identified for BA.5+MP vs. BA.5 (**Figure 5B**).

Another way of representing such data is by heat maps (46). When the top 30 cytokine USRs (by absolute z score) for BA.5 vs. PBS were ranked, and shown next to the aforementioned z scores for CRS and the Classical signature, there were no significant positive correlations (**Figure 5C**, Pearson correlations). The correlation was also poor for comparisons with the mean cytokine USR z scores previously generated from 4 human studies of SARS-CoV-2 infected lung tissues (46) (**Figure 5C**, Bishop, mean human, p=0.51). These poor correlations (**Figure 5C**) likely again reflect the relatively mild disease seen in the BA.5 mACE2-hACE2 mouse model.

When the top 30 cytokine USRs for BA.5+MP vs. BA.5 were ranked and compared with z scores from CRS in COVID-19 infected patients and the Bishop et al., 2022 study, significant positive correlations again emerged (**Figure 5D**). As in **Figure 5B**, there was no significant correlation with the classical signature (**Figure 5D**).

Taken together these analyses suggest that MPs in the SARS-CoV-2-infected lung pushed the cytokine signatures towards a CRS profile. However, MPs did not induce overt clinical disease in the BA.5-infected mice, nor were we able to detect significant histological changes in lung sections (**Supplementary Figures 2B**, **5**).

### MPs promote expression of Hsp70 genes during SARS-CoV-2 infection

At the top of the DEG lists for BA.5+MP vs. BA.5 are a number of genes that are associated with stress, and/or genes that have previously been identified as being induced after smoke or diesel particle inhalation, or in other lung diseases/disease models (**Table 1**). The top DEGs for BA.5+MP vs. BA.5 at 6 dpi where heat shock protein 70 (Hsp70) family members Hspa1a and Hspa1b, with Hsp70 induction well described in the MP literature (see Discussion).

**Table 1:** Many top up-regulated DEGs are associated with stress responses and are also identified in other lung diseases or disease models.

Gene	Log_2_FC 2 dpi	Log_2_FC 6 dpi	Function	Regulation/activity in lungs
Hspa1b	–	2.64^1^ (1)	Stress-induced transcription chaperones. Cytoprotection (Hsp70 family members)	Biomarker for bronchopulmonary dysplasia (122). Upregulated in ARDS model (123). Promotes epithelial cell repair (124)
Hspa1a	–	2.46^1^ (2)		
Cxcl5	–	2.28^1^ (3)	Chemokine. Tissue remodelling, neutrophil^2^ recruitment.	Correlates with lung function decline in COPD patients and mouse smoking model (125)
Nr4a1, nuclear receptor subfamily 4 group A member 1	1.52^1^ (1)	1.77 (5)	Nuclear receptor with transcription activator activity.	Induced by acrolein (smoke chemical) in A529 cells (126).
Ccn1	1.43^1^ (2)	1.33 (6)	Matricellular protein; inflammation, tissue repair.	Associated with ARDS severity (127)
Cxcl1	–	1.09 (9)	Chemokine. Wound repair, neutrophil^2^ recruitment.	Induced by diesel exhaust in mouse model (128)
Egr3	1.03 (10)	1.07 (10)	Immediate early stress response gene	Upregulated by acrolein (126). Upregulated in lungs from COPD patients (129)
Atf3 Activating transcription factor 3	0.54 (51)	0.94 (14)	A master regulator of stress responses	Induced in lungs by particulate matter (130). Key role in lung regeneration (131).
Klf2, Krüppel-like Factor 2	0.82 (14)	0.89 (16)	Transcription factor expressed by multiple cell types in the lung	Induced by air pollution (twin study) (132). (see also **Supplementary Figure 6**)

Top up-regulated DEGs on 2 and 6 dpi for BA.5+MP vs BA.5 (**Supplementary Tables 5**, **6**). Log_2_FC are shown, with “ - “ indicated if the gene is not a DEG on that day. The numbers in brackets [e.g. (1)] represent the position in the DEG list sorted by fold change, i.e. Hspa1b is the most up-regulated DEG at 6 dpi. ^1^Genes that were synergistically induced for BA.5+MP vs BA.5 such that the cpm ratios (BA.5+MP/(BA.5 plus +MP) were >1 (**Supplementary Table S8**). ^2^No neutrophil-associated signatures were identified in any of the bioinformatic analyses, suggesting that in this setting this activity did not manifest.

We also calculated which genes were synergistically induced (i.e. genes for which the cpm for BA.5+MP > +MP alone plus BA.5 alone). Hspa1a and Hspa1b emerged as the most synergistically induced genes, with Cxcl5, Nr4a1 and Ccn1 also identified (**Table 1**; **Supplementary Table 8**). RT-qPCR was used to validate differential expression of Nr4a1, Ccn1, Hspa1a and Hspa1b, with log_2_ fold-changes and statistical significance consistent with the RNA-Seq data (**Supplementary Figure 4B**).

### Overlap of DEGs

When the upregulated DEGs for BA.5+MP vs. BA.5 were compared with upregulated DEGs for +MP vs. PBS, only a small number of genes were found to be common to both DEG lists at both time points (n=12 for 2 dpi and n=2 for 6 dpi) (**Supplementary Figure 6**). Thus MPs on their own induced a largely different set of genes, when compared with MPs in a SARS-CoV-2 infection setting. This observation supports the contention that the detrimental activity of MPs might best be observed in the dysregulation of the inflammatory processes during the course of a disease (45), rather than as an imposition of fixed MP-specific responses.

Despite the overall low level of overlaps, treatment with MPs was consistently associated with upregulated of one DEG, kruppel-like factor 2 (Klf2) (**Supplementary Figure 6**).

## Discussion

We show herein in a mild disease model of SARS-CoV-2 infection and disease (omicron BA.5 infection of mACE2-hACE2 mice) that MP inoculation into the lungs dysregulated the innate inflammatory responses against the virus. MPs in the SARS-CoV-2 infected lungs lead to depressed innate proinflammatory immune responses at 2 dpi, with an elevated innate proinflammatory profile identified at 6 dpi. The latter profile showed significant correlation with the ‘cytokine release syndrome’, which is a potentially lethal manifestation of severe COVID-19 (117, 133). Thus the influence of MPs might be viewed as moving the inflammatory response away from protective inflammation (134, 135) toward pathological inflammation. However, despite this modulation, MP-mediated influences on SARS-CoV-2-induced disease was clinically inapparent in this model, with no overt clinical or histologically-detectable changes observed.

The ability of MPs to block efferocytosis via binding to the efferocytosis receptor Tim4 (42), may provide a basis for understanding at least some of the transcriptional perturbation described herein. Specifically, the clearly depressed phagocytosis signatures, the identification of multiple annotations associated with modulation of macrophage responses (with macrophages the key mediators of efferocytosis), and the overall lack of detectable changes to adaptive immune responses, might be viewed as consistent with a role for dysregulated phagocytosis/efferocytosis (115, 116). Reduced phagocytosis of SARS-CoV-2 infected cells at 2 dpi might reduce M1 macrophage activation, subsequent pDC activation (85), and the ensuing cytokine responses (86, 136). Less phagocytosis at the peak of infection might also lead to more secondary necrosis and/or necroptosis of SARS-CoV-2 infected cells, thereby promoting inflammation at 6 dpi (137, 138).

Inhibiting phagocytosis/efferocytosis may not be the only mechanism in play, as MPs alone provided some stress-associated, damage and pro-inflammatory signatures (**Figure 1**), which may also influence the SARS-CoV-2-mediated innate responses in the lung. The top DEGs for BA.5+MP vs. BA.5 support this contention as some of these DEGs have also been identified in studies of smoke or diesel particle inhalation (**Table 1**). The top DEGs for 6 dpi were heat Hsp70 family members, Hspa1a and Hspa1b, with these genes also synergistically induced (**Table 1**; **Supplementary Table 8**). Hsp70 up-regulation is reported in a range of MP exposure settings including mussels (139), goldfish (140) and *Daphnia* (141). Hsp70 stress responses are involved in a vast range of pathologies (142) and play a role in cytoprotection against environmental challenges (122) including SARS-CoV-2 infection (143). The top cytokine USR by z score for +MP vs. PBS was IL-1β, a cytokine matured and released via inflammasome activation (**Figure 1**). Our observation thus support a recent speculation that MPs might activate the inflammasome (144), with IL-1β also a dominant signature at 6 dpi for BA.5+MP vs. BA.5.

Nr4a1 (**Table 1**) was recently identified as a marker of a subset of group 2 innate lymphoid cells (ILCs) (145), with ILCs implicated in our previous study of MPs and a viral arthritis model (45). However, we have been unable to find a compelling signature that implicates ILCs as important players in the current setting. Nr4a1-dependent CD16.2^+^ monocytes have been implicated as potential precursors of CD206^–^ interstitial macrophages (98, 99, 146). However, changes in interstitial macrophages were identified for +MP vs. PBS (**Figure 1**), where Nr4a1 was not a DEG, but interstitial macrophages were not identified for BA.5+MP vs. BA.5 (**Figures 3**, **4**) where Nr4a1 was a top DEG (**Table 1**). Conceivably, the up-regulation of this gene is associated with lung epithelial cells, where it has been identified as a novel allergy-associated gene (147).

Klf2 was the only DEG upregulated by MPs in all settings and time points studied herein (**Supplementary Figure 6**). Klf2 is upregulated in blood endothelial cells as a result of sheer stress (148), but is ordinarily down-regulated during SARS-CoV-2 infection (149, 150). How these activities might be relevant in the current context is unclear. Klf2 upregulation may be related to the general dysregulation of macrophage activities by MPs seen herein, with Klf2 expression associated with alveolar macrophage self-renewal (151) and inhibition of M1 polarization (152).

Although many reports suggest MPs induce ROS (40, 41), we did not identify an increase in ROS as a major consequence of MP exposure, with the MPs used in this study being free of azide (39). IPA Disease and Functions annotation actually providing a slightly negative z score for “Production of reactive oxygen species” (**Supplementary Table 1**). MP-induced changes to the lung microbiota (37) might also provide a potential mechanism for the modulation of innate responses; however, our metagenomics analysis failed to identify any changes in the microbiome (**Supplementary Figure 7**).

This study has a number of limitations; we have not investigated the activity of MPs with different shapes, sizes, compositions and leachates (153, 154). However, such considerations give rise to an unworkably large number of experimental variables that are beyond the scope of this foundational study. We have also not examined longer exposure times or different exposure doses, and we have also only examined the consequences of MPs in one mild mouse model of COVID-19. However, the mild MP-associated transcriptional changes seen herein (**Figure 1**) might be drowned in a more severe model of COVID-19, where infection-induced gene expression changes are substantially more widespread and robust (46).

In summary, we provide herein evidence that MPs in the lung can dysregulate innate inflammation-associated transcriptional responses to SARS-CoV-2 infection in a mouse model of mild COVID-19. However, the dysregulation did not result in overt changes in disease or histopathology, suggesting MP-mediated changes were generally mild. To what extent MPs might influence COVID-19 disease severity at a population level may warrant investigation. An approach might be to compare matched populations exposed to high (155) and low levels (156) of airborne MP pollution, although separating the influence of MPs from other factors may represent a formidable challenge. Nevertheless, such analyses are conceivable with, for instance, mortality associated with fine particulate matter air pollution from coal powered electricity plants was recently estimated (157).

Considerable speculation surrounds the potential health impacts of MP inhalation by the general public (21, 158–160), with limited compelling *in vivo* data from human studies and animal models. However, an emerging theme from such studies, including the current report, is that MPs can dysregulate and/or promote inflammatory processes in specific disease settings (15, 45, 161–164). Future animal research in this area would benefit from use of realistic MP doses; although we need (i) better insights into what such doses actually are in different human populations, and (ii) adoption of standard units for MP exposure (165) (e.g. µg/kg/d) so that studies can be compared and dose effects understood. Avoidance of artefacts associated with preservatives such as azide is clearly also critical for future meaningful medical research in the MP space (39). Future animal research might also examine the effect of chronic MP exposure using multiple doses, as well as comparing the effects of different MP sizes, shapes, compositions and leachates.

## Data availability statement

The datasets presented in this study can be found in online repositories. The names of the repository/repositories and accession number(s) can be found below: PRJNA1036279 (SRA).

## Ethics statement

The studies involving humans were approved by the QIMR Berghofer Medical Research Institute Human Research Ethics Committee (P3600). The studies were conducted in accordance with the local legislation and institutional requirements. The participants provided their written informed consent to participate in this study. The animal study was approved by QIMR Berghofer Medical Research Institute animal ethics committee -P3600. The study was conducted in accordance with the local legislation and institutional requirements.

## Author contributions

CB: Writing – review & editing, Writing – original draft, Visualization, Methodology, Formal analysis, Data curation. KY: Writing – review & editing, Investigation. WN: Writing – review & editing, Formal analysis. DR: Writing – review & editing, Supervision, Methodology, Funding acquisition. BT: Writing – review & editing, Investigation. TL: Writing – review & editing, Investigation, Formal analysis. AS: Writing – review & editing, Writing – original draft, Visualization, Supervision, Project administration, Methodology, Funding acquisition, Data curation, Conceptualization.
